# Structural changes within the bifunctional cryptochrome/photolyase *Cr*aCRY upon blue light excitation

**DOI:** 10.1038/s41598-019-45885-7

**Published:** 2019-07-09

**Authors:** Sophie Franz-Badur, Alexander Penner, Simon Straß, Silke von Horsten, Uwe Linne, Lars-Oliver Essen

**Affiliations:** 1 Unit for Structural Biochemistry, Department of Chemistry, Philipps University Marburg, Hans-Meerwein Straße 4, 35032 Marburg, Germany; 2 Synovo GmbH, Paul-Ehrlich-Straße 15, 72076 Tübingen, Germany; 3 LOEWE Center of Synthetic Microbiology, Philipps University Marburg, Hans-Meerwein Straße 4, 35032 Marburg, Germany

**Keywords:** Proteins, Structural biology

## Abstract

Cryptochromes (CRYs) are an ubiquitously occurring class of photoreceptors, which are important for regulating the circadian rhythm of animals *via* a time-delayed transcription-translation feedback loop (TTFL). Due to their protein architecture and common FAD chromophore, they belong to the same superfamily as photolyases (PHLs), an enzyme class that repairs UV-induced DNA lesions upon blue light absorption. Apart from their different functions the only prominent structural difference between CRY and PHL is the highly variable C-terminal extension (CTE) of the former. The nature of the CTE is still unclear and highly speculated. In this study, we show by hydrogen/deuterium exchange and subsequent mass-spectrometric analysis that the CTE of the animal-like cryptochrome from the green algae *Chlamydomonas reinhardtii* (*Cr*aCRY) binds to the surface of the photolyase homology region, which flanks the DNA binding site. We also compared the fully oxidized and fully reduced states of the flavoprotein and designed a tool, so called light chamber, for automated HDX-MS measurements of photoreceptors in defined photostates. We could observe some striking differences between the two photostates and propose a model for light-dependent switching of this bifunctional cryptochrome.

## Introduction

Trapping dynamic structural changes of a protein between its active/catalytic and inactive/resting state is a difficult task for scientists looking for structure-function relationships. In the last years, several time-resolved methods have been developed, which provided insight into time-dependent structural changes of various photoreceptors, e.g. bacteriorhodopsin (Nango *et al*.^1^, TR-SFX) and bacteriophytochrome (Takala *et al*.^2^, TR-SAXS). These methods are highly promising, but still expensive and not easily accessible to most users as they rely on advanced synchrotrons and X-ray free electron lasers. Accordingly, to compare different states of a protein, hydrogen/deuterium exchange mass spectrometry (HDX-MS) has attracted attention^3–6^, because HDX-MS is a relatively fast method and allows a distinct characterization of structured and unstructured areas of a protein sample in solution. For this the relative deuterium uptakes of peptide fragments are compared, as the exchange rates of structured and unstructured regions significantly differ. Furthermore, this method can also identify the binding site of a substrate or a reaction partner in solution.

In this study we focused on light-induced and redox-state dependent structural changes of the animal-like cryptochrome from the green alga *Chlamydomonas reinhardtii* (*Cr*aCRY)^7,8^. Apart from light-insensitive mammalian cryptochromes, cryptochromes are generally flavin-comprising photoreceptors, which monitor available daylight by a flavin adenine dinucleotide (FAD) chromophore that is bound non-covalently within the C-terminal domain of the photolyase-homology region (PHR)^9^. These photoreceptors are found in all three kingdoms of life including animals. Until now, four different types have been assigned^10,11^, for example two major and highly related groups are found in insects (type 1)^12^ and mammals (type 2)^13,14^. The animal cryptochromes (CRY) differ in term of their *in vivo* functionality, because type 1 CRY act as blue-light photoreceptors for resetting the circadian clock. In contrast, type 2 CRY lack any known photosensory function^15^, although they are still crucial components of the core of the circadian clock. Two other less related types of cryptochromes include the plant cryptochromes as well as the DASH-type cryptochromes. The latter are still capable of single-stranded DNA repair^16^. In terms of their structural features, cryptochromes are highly related to (6-4) photolyases for type I and II CRY and to class I CPD photolyases for plant and DASH CRY. A common feature that makes them distinct from photolyases is given by their elongated C-terminal extensions (CTEs), which adopt variable lengths. For example, the length of a cryptochrome’s CTE varies from 23 amino acids (aa) in the type I cryptochrome of *Drosophila melanogaster* (*Dm*CRY)^17^ to 191 aa in CRY1 from the plant *Arabidopsis thaliana*^13^ and more. The role of the CTE in affecting the photoreceptor function of CRY is still elusive, as there is only just one example of a successfully analyzed structure of a cryptochrome with CTE, namely *Dm*CRY^17^. In that case, the CTE is short, 23 aa, and wound up to an α-helix that binds to the FAD binding domain. Busza *et al*. and Dissel *et al*. showed, that a CTE-truncated *Dm*CRY mutant is a constitutively active photoreceptor and capable to bind TIM, another circadian clock component present in animal cells, without light as strongly as the wild-type *Dm*CRY which is exposed to light^18,19^. However, the CTE-deficient mutant binds to TIM already in the dark, whereas the wild-type (WT) cryptochrome requires light to enable it for recruiting TIM^20,21^. This lead to the conclusion, that the CTE undergoes a light-dependent conformational change to unblock the TIM binding site in *Dm*CRY^22^. Given the light-dependent binding of Jetlag (JET) to *Dm*CRY^23^, Ozturk *et al*. proposed a mechanism combining DmCRY, TIM and JET-dependent degradation^24^. For the light-induced change in *Dm*CRY, Ozturk *et al*.^23^ and Ganguly *et al*.^25^ proposed changes of the hydrogen-bonding pattern and protonation state, respectively, around the FAD binding pocket, which result in triggering a movement of the CTE.

The *Dm*CRY ortholog from *C*. *reinhardtii*, *Cr*aCRY, was shown to regulate the transcription of various genes by different light qualities^7^ and plays a significant role in the sexual life cycle of *C*. *reinhardtii* together with a plant-like CRY and a phototropin ortholog^26–28^. Interestingly, given the close ontogenetic relationship between type 1 CRY and (6-4) photolyases, *Cr*aCRY demonstrates not only a regulatory function but is also capable to repair DNA comprising (6-4)-pyrimidone-pyrimidine lesions, (6-4)PP^8^. The CTE of *Cr*aCRY is considerably longer than that of *Dm*CRY by comprising 99 aa, which are 17% of the overall enzyme. The structure of the PHR domain has been recently solved by X-ray crystallographic analysis^8^, but the WT protein generated no crystals so far. Accordingly, no structural information on the long CTE of *Cr*aCRY is available and the role of the C-terminus is hence uncertain. Oldemeyer *et al*. proposed that the WT of *Cr*aCRY forms a heterodimer involving the CTE^29^ in the dark state, which we should be able to observe using HDX-MS data.

## Results and Discussion

**Figure 1:**
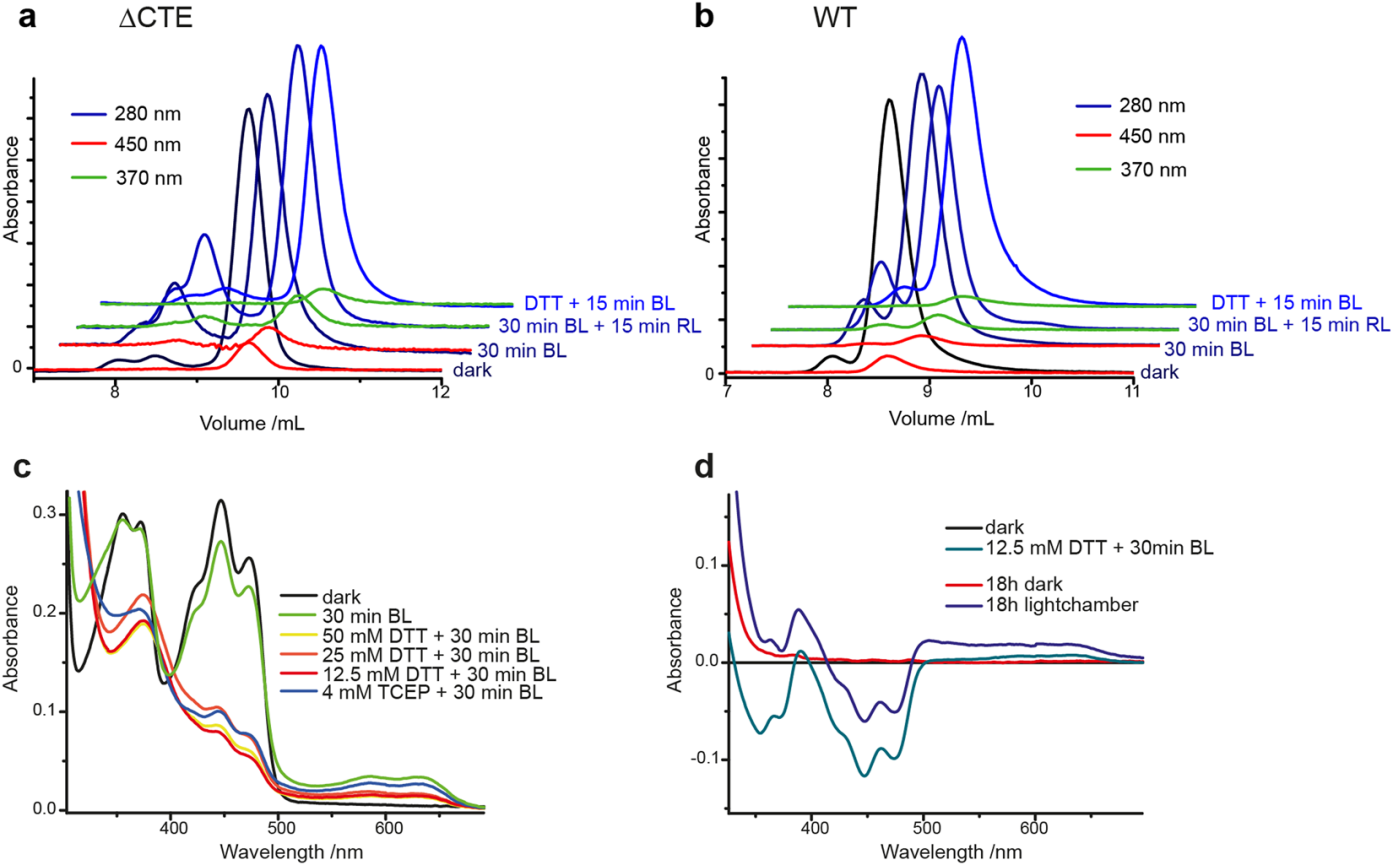
Analytical size exclusion of *Cr*aCRY-∆CTE (**a**) and WT (**b**). In both cases most (80–97%) of the protein sample elute in the monomeric state. (**c**) UV/Vis measurement of *Cr*aCRY WT treated with blue light (BL) and different concentrations of dithiothreitol (DTT) or tris(2-carboxyethyl)-phosphine (TCEP). The FAD cofactor was reduced to FADH^−^ under all conditions (except only BL), but in the sample treated with 12.5 mM DTT less degradation can be observed. (**d**) Difference spectra of the photoreduction of *Cr*aCRY WT with 12.5 mM DTT and 30 min BL as well as reoxidation of FADH^−^ in- and outside the light chamber. After 18 h the sample outside the light chamber entirely reoxidized to FAD_OX_ (red line), whereas for the sample inside the chamber 70% remain in the FADH^−^ state with some contributions from FADH° and FAD_OX_ (blue line).

The purified *Cr*aCRY samples were examined by analytical size exclusion chromatography (SEC) under different illumination conditions. We were expecting most of the WT sample to be in the dimeric state according to Oldemeyer *et al*.^29^, but our SEC measurements differ significantly, as most of the protein in *Cr*aCRY samples remain in the monomeric state (Fig. 1a,b). Samples were analyzed before SEC *via* UV/Vis spectroscopy to check the redox state of the FAD (Supplementary Fig. S1). Over 93–96% of the fully oxidized as well as the fully reduced state in both variants eluted as monomeric species (Supplementary Table S1). In contrast, SEC data of *Cr*aCRY generated in its semiquinoid state by pre-illumination indicate the formation of species with a somewhat bigger size, which could be due to dimerization, structural change or (partial) unfolding of the protein. As the oxidized and fully reduced *Cr*aCRY species lack any absorption at 370 or 450 nm for the dimer-like SEC peak, these species might be caused by a contamination of a misfolded form of *Cr*aCRY lacking any bound FAD. By calculating the percentages of dimer-like and monomeric species over the whole chromatogram traces at different wavelengths (Supplementary Table S1), *Cr*aCRY WT and the truncated ΔCTE variant in their semi-quinoid states show an almost invariant 1:1 incorporation of FAD for the observed dimer-like species. The discrepancy between our SEC data and the data published before indicating higher oligomeric species could be due to differences of the *Cr*aCRY purification scheme. In our improved purification protocol for crystallization and HDX analysis, we used DNase I as well as a heparin affinity column to remove any remaining (oligo)nucleotides that may stuck to the protein as *Cr*aCRY was shown before to bind DNA and repair (6-4)PP DNA damages^8^.

To compare structural changes of *Cr*aCRY in its oxidized (FAD_OX_/dark) and fully reduced (FADH^−^/light) state by HDX-MS analysis, a suitable buffer condition for HDX and a reducing agent which keeps the fully reduced state stable over several hours without denaturing the protein had to be determined. A Tris-buffer (pH 7.8) with a low salt concentration and without any glycerol was chosen for HDX-MS analysis; under these conditions photoreduction with various concentrations of dithiothreitol (DTT) and tris(2-carboxyethyl)phosphine (TCEP) was observed (Fig. 1c). For 12.5 mM DTT a significant peak at 376 nm and a local minimum of absorption at 344 nm could be detected. The five characteristic absorption peaks^11^ for FAD_OX_ are clearly diminished. As the fully reduced state rapidly reoxidizes in the dark and under aerobic conditions, we developed a light chamber, which fits the specific requirements of the experimental set-up (Supplementary Fig. S2). In difference-absorption spectra, a clear contrast between a sample, which was kept after photoreduction in the dark, and a sample that was kept in the light chamber can be seen (Fig. 1d). After 18 h in the light chamber, the protein still shows the typical absorption of the fully reduced FADH^−^. The HDX-MS measurements for *Cr*aCRY and the deletion variant *Cr*aCRYΔCTE that lacks the C-terminal extension (497–595) and hence comprises only the photolyase-homology region (PHR, 1–496) were performed using the determined conditions.

**Figure 2:**
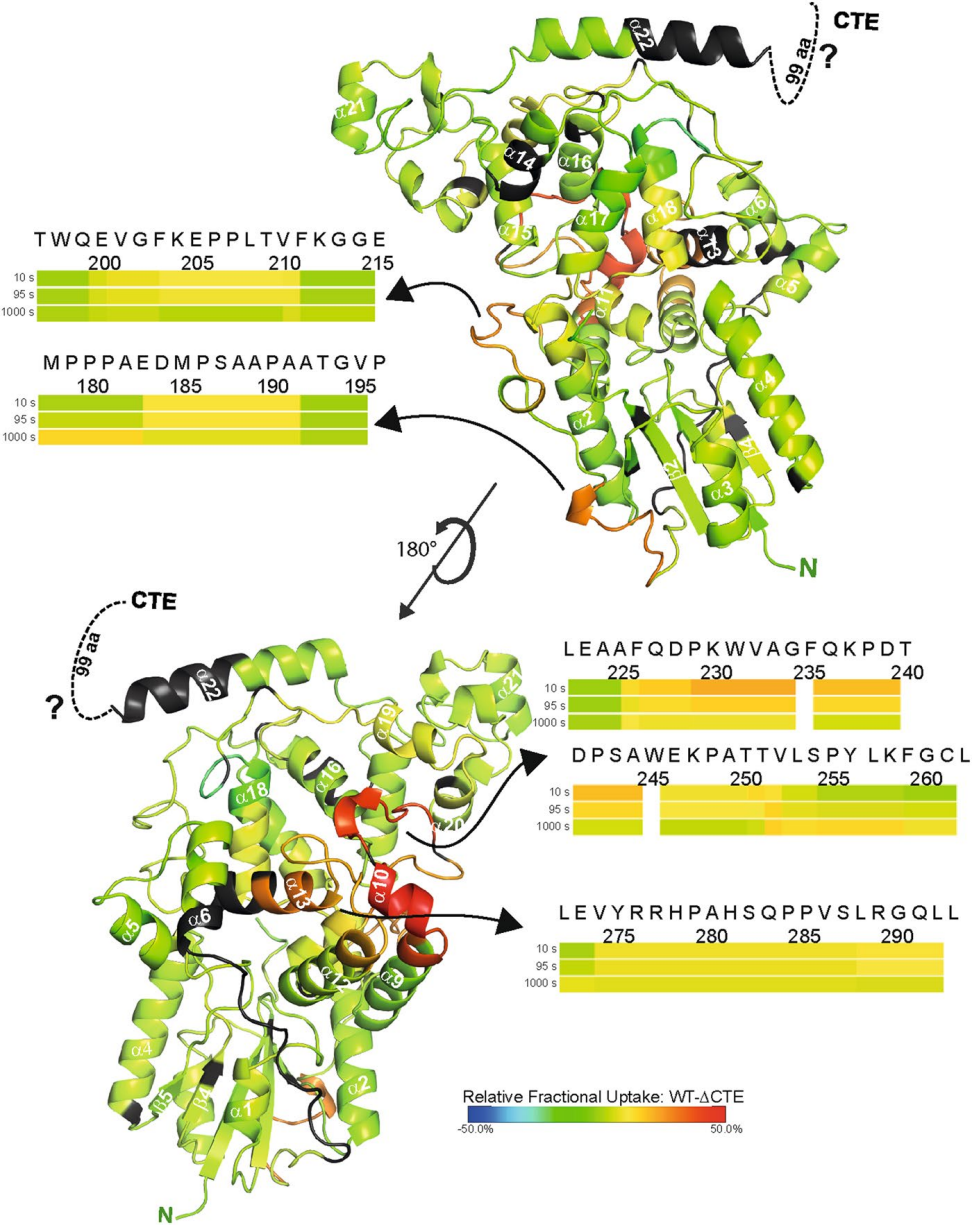
Selected heat maps of the relative fractional deuterium uptake of *Cr*aCRY-WT minus *Cr*aCRY∆CTE. The relative uptake of the FAD_OX_ state at 10 s was mapped onto the *Cr*aCRY∆CTE structure (PDB: 5ZM0). Red sections correlate with a higher deuterium uptake in full-length *Cr*aCRY, while blue sections correlate with a higher uptake in the ∆CTE variant than in WT. For the black segments no peptides could be assigned by HDX-MS analyses. Changes were observed for the linker between the N- and C-terminal domains and for a helix bundle in the C-terminal domain (α10 to α13) that is exposed at the protein surface. This correlates to a higher flexibility or solvent accessibility of these regions in the WT.

**Figure 3:**
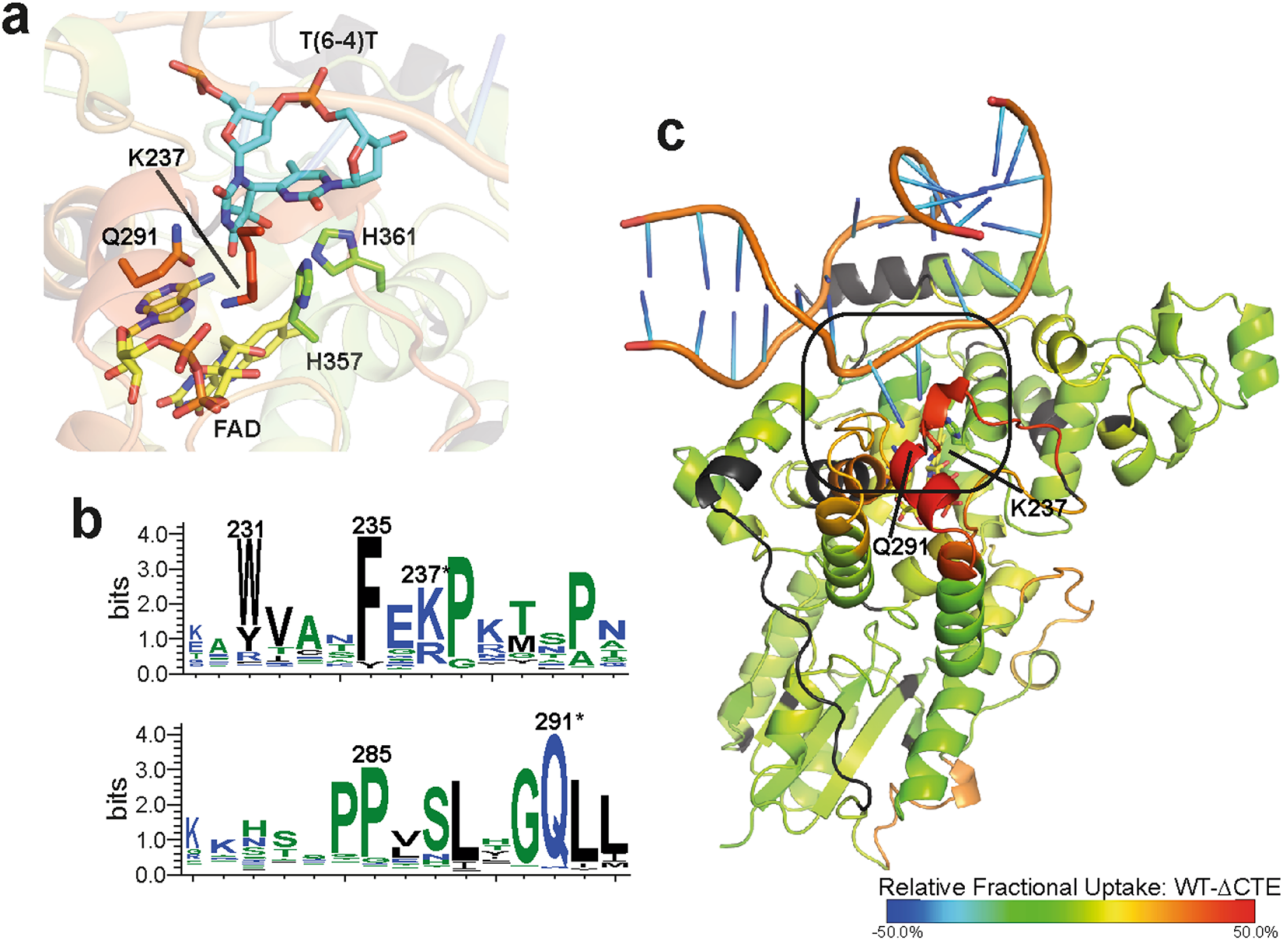
Closer view on the DNA binding site of *Cr*aCRY. For that the relative uptake of the FAD_OX_ state at 10 s was mapped onto the *Cr*aCRY∆CTE-DNA structure (PDB entry: 6FN0). (**a**) Here, the (6-4) damaged DNA (blue) is bound to the enzyme and the two catalytic histidines (green) are shown. (**b**) Q291 and K237 play a crucial role in DNA binding and are highly conserved (marked with *) as shown in the WebLogo that was generated from a multiple sequence alignment of animal-like cryptochromes^8^. Both residues are located on a stretch with high deuterium uptake in the WT. (**c**) The overall binding mode of the dsDNA is expected to be unaffected between *Cr*aCRY-WT and *Cr*aCRY∆CTE when taking the HDX analysis results without DNA into account.

After running the ProteinLynxGlobalServer software (*Waters*) and importing search results into DynamX (*Waters*), 132 peptides could be identified for *Cr*aCRY yielding 75% coverage at an overall redundancy of 3.43. For the CTE, only four peptides could be found in the *Cr*aCRY dataset with almost no overlaps. Therefore, for further analysis we focused on the PHR domain. Comparing the WT with the truncated ∆CTE, four regions with significant differences in deuterium uptake can be found in the dark aka FAD_OX_ (Fig. 2, Supplementary Fig. S3) and light aka FADH^−^ samples (Supplementary Table S2). When the dark data are mapped onto the structure (PDB entry: 5ZM0, Fig. 2) of *Cr*aCRY∆CTE, two loop regions (184–192; 200–211), both located at the long connecting loop between the N-terminal Rossman-like domain and all-α C-terminal domain, are showing a higher uptake in the WT sample. By rotating the protein by 180°, another region with significant difference can be depicted. Here, a bundle of five helices oriented towards the protein surface with α10 on the top display a much greater uptake in the WT protein. Namely, peptides covering regions between 225 and 252 as well as between 274 and 293 exhibit the greatest variation. These four identified regions demonstrate the same behavior in light and dark samples. Accordingly, these features are independent of the redox state of *Cr*aCRY. The only difference is the missing C-terminus. So how can we interpret these data? A higher uptake indicates either a more flexible or easily accessible region in one sample, but a hidden region, maybe by dimerization or interaction, in the other sample. We already know that the ∆CTE variant is monomeric under the used HDX-MS conditions like full-length *Cr*aCRY, so a dimerization site is unlikely. Both proteins are able to bind and repair DNA^8^, but during purification DNA contaminants were removed and both samples were treated exactly the same. This leads to the conclusion, that in the presence of the CTE, the identified regions underlie a structural change. Looking at a multiple sequence alignment of over 500 sequences (described in Franz *et al*.^8^) of animal-like cryptochromes and (6-4)photolyases, the helix bundle exposes several highly conserved residues. Two of them, K237 and Q291, are involved in formation of the DNA binding site (Fig. 3). We propose, that this binding site might become partially unfolded or somehow changes it’s dynamics in the presence of the CTE so it becomes more accessible to the deuterium in the HDX experiment. Such non-canonical, increased deuterium exchange rates upon ligand binding to a protein have been described in the past, e.g. for oxy- and deoxy-myoglobin, and designated as type-2 scenarios^30^.

**Figure 4:**
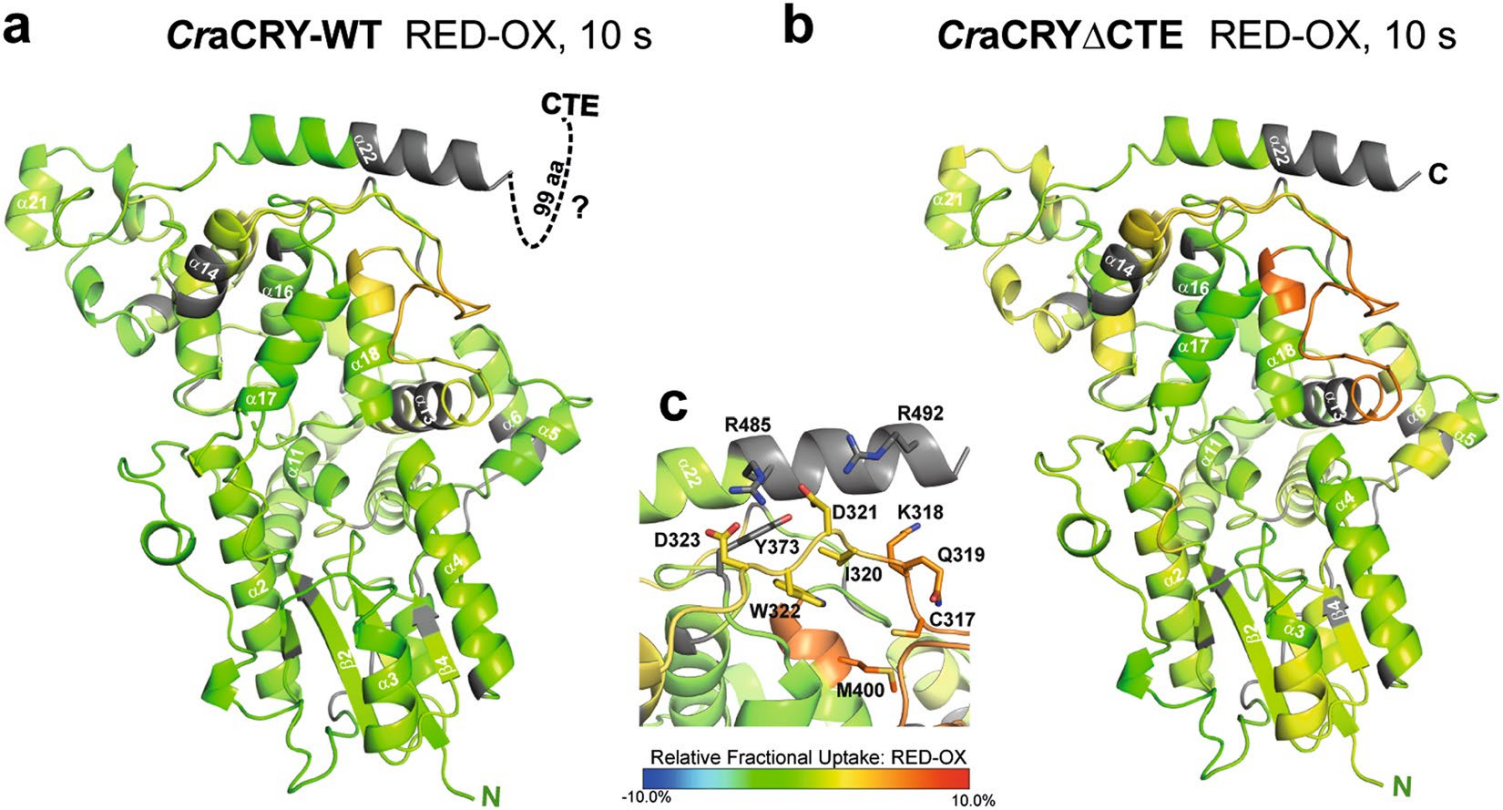
Relative fractional deuterium uptake at 10 s of the reduced (FADH^−^) minus the oxidized (FAD_OX_) states of (**a**) *Cr*aCRY-WT and (**b**) truncated *Cr*aCRY∆CTE, both are mapped onto the *Cr*aCRY∆CTE structure (PDB: 5ZM0). A highly significant difference can be observed in a loop region close to the C-terminal helix α22. **c**) The shown loop (300–326) presenting the distal tryptophan, W322, is part of the highly conserved tryptophan triad in photolyases and cryptochromes. W322 is flanked by the polar residues D323 and D321, which form a network of hydrogen bond interactions with the basic residues R485 and R492 from the C-terminal α22 helix. This charged network shields the distal electron donor of the electron transfer cascade, Y373, from exposure at the protein surface. The 320–326 loop exhibits a higher deuterium uptake in the reduced state, which correlates with higher flexibility. We suggest that the tight interaction between D323/D321 and R485/R492 gets disrupted upon formation of the Y373° radical, so that the α22 helix loses conformational restraint by interaction with the PHR.

**Figure 5:**
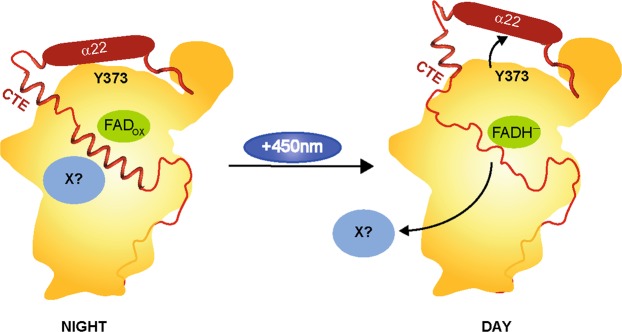
Model of the structural movement in *Cr*aCRY from the fully oxidized to the hydroquinone state of the FAD based on the HDX data. In between, the neutral semiquinone state is formed (FADH°), but we have no structural information addressing this state. In the shown model, the C-terminal extension (CTE, red) is located over the protein surface and is partly structured. We propose that an unknown signaling partner X (light blue) can bind to *Cr*aCRY during the night, when it is located over the whole cell body. During the day, the hydroquinone state is forming (FADH^−^). Because of the radical pair formation [Y373° and FADH°] during photoreduction, the H-bond network between α22, D323 and D321 is disrupted which leads to a movement of the helix and therefore a change in the CTE. The structured region in the CTE gets unfolded and factor X is getting released.

Comparing the dark (FAD_OX_) and light (FADH^−^) states of WT and the ∆CTE variant, two regions with significant differences in the uptake can be identified (Fig. 4, Supplementary Fig. S4). The differences can be assigned to a loop between helix α13 and helix α14 (300–326) as well as a short part of helix α18 (399–403) pointing towards this loop (Fig. 4a,b). Looking at the specific residues located in this region, one of the essential tryptophans of the highly conserved tryptophan triad, the distal W322, can be found on the loop (Fig. 4c). In PHLs and CRYs this tryptophan triad is responsible for the efficient reduction and stabilization of the semi-reduced (FAD°^−^ or FADH°) or fully reduced (FADH^−^) state of the flavin cofactor. *Cr*aCRY and other animal CRYs (type 1) and also 6-4 photolyases show an elongation of this tryptophan triad by a fourth aromatic residue, either tyrosine or another tryptophan. After flavin excitation of *Cr*aCRY, an electron is transferred from the proximal tryptophan W399 to FAD, then the electron hole gets filled by the medial tryptophan W376, after that the distal W322 and finally by Y373 as fourth aromatic residue, which forms a relatively stable radical close to the protein surface^8,29,31^. Interestingly, Y373 is not covered by HDX-MS, but the residues D321 and D323, which form a network of charged residues together with R485 and R492 located on helix α22, are also located on the described loop region. As the relative fractional uptake from the reduced state minus the oxidized state is significantly higher for this region, this can be interpreted as a conformational change of the stretch involving these residues from the oxidized to the reduced state. We therefore propose that after the excitation of the flavin and formation of the Y373° radical, the van-der-Waals bond network between D321, D323, Y373, R485 and R492 changes and triggers an overall movement of the loop (300–326). From our analysis, we are not able to show if α22 is moving in some way, as it is only partly covered, but this possibility is not excluded. Also a part of α18, which is in close contact with the relevant loop, seems to be effected from the movement. By comparison of both relative uptake maps to each other, the changes of deuterium uptake in the truncated sample and in consequence the movement of this specific region are greater. This can be explained by the CTE allowing movement of α22 only to some extent (Fig. 5). It was shown before that *Cr*aCRY is enriched in the nucleus during the day and delocalized over the whole cell in the night^28^. The proposed mechanism might explain the bifunctional role of *Cr*aCRY, *e*.*g*. by acting as a photolyase during the day and binding/releasing a signaling partner X during the night phase. The yet unknown partner X could be the plant CRY of *C*. *reinhardtii*, as *Cr*aCRY and *Cr*pCRY have been supposed to form a complex in the dark which results in a loss of mating ability and thus the formation of inactivated gametes^28^.

In conclusion, we were able to develop a specific tool to keep photoreceptors in their different photostates during HDX-MS analysis and used it to perform measurements of *Cr*aCRY with and without its CTE. We gained first insights of the structural changes the cryptochrome undergoes upon photoreduction and are a step closer to address the question of how this photoreceptor can perform both DNA repair and regulation of gene transcription.

## Materials and Methods

### Design of the light chamber

The light chamber was designed and manufactured in cooperation with the precision mechanics workshop of the chemistry department at the *Philipps University of Marburg*. A detailed schematic representation can be found in the supporting information. The built-in LEDs 450 nm (5.2 cd, ELD-450–525), 625 (60–70 cd, 5RAA5111P) and 735 nm (7.5 cd, ELD-720–524) where purchased from *Roithner LaserTechnik GmbH*. The three sample holders can be programmed separately with two different illumination intervals. Either a long interval with 30 s of illumination and 270 s darkness or a short interval with 5 s of light and 20 s in the dark. The rechargeable battery has a capacity of 2600 mAh and can be charge over a micro-USB port.

### Sample preparation

*Cr*aCRY-WT and *Cr*aCRY∆CTE constructs were expressed and purified following published procedures (Beel *et al*., Franz *et al*.^7,8^) with a heparin column before size exclusion chromatography to remove any DNA contaminants. For HDX-MS, the protein was transferred via PD-10 into a low salt buffer (10 mM Tris, 100 mM sodium chloride, pH 7.8). To produce the fully reduced FADH^−^ state, 12.5 mM DTT was added and the samples were illuminated for 30 min with a high power blue light LED (9.7 mW cm^−2^ at a distance of 10 cm, Roithner Lasertechnik).

### Analytical size exclusion chromatography

After purification, ∆CTE (**a**) and WT (**b**) samples were analyzed with a Superdex 75 increase 10/300 GL which was calibrated at 4 °C at 0.5 mL/min (y = −0.15642x + 3.29765) prior to use. 200 µL sample (c = 2 mg/mL) was injected at the column and elution was performed in phosphate buffer (50 mM sodium phosphate, 100 mM sodium chloride, pH 7.8). The absorption was detected with a multiwavelength detector. Samples were treated before with blue light (BL) or in combination with red light (RL) or with DTT.

### Steady-state UV/Vis spectroscopy and photoreduction assay

Absorption spectra of *Cr*aCRY variants were recorded using a V-660 spectrometer (JASCO). The protein solutions were measured in a low salt buffer (10 mM Tris, 100 mM sodium chloride, pH 7.8) with varying concentrations of dithiothreitol (DTT) and tris(2-carboxyethyl)phosphine (TCEP). Spectra were recorded after different illumination times using a high power LED (λ_max_ = 450 nm; 9.7 mW cm^−2^ at a distance of 10 cm, Roithner Lasertechnik) at 2 °C. To simulate a HDX-MS measurement samples were put in the light chamber, which was cooled at 4 °C.

### Hydrogen-Deuterium-Exchange-Mass Spectrometry (HDX-MS)

To promote the light state formation, 12.5 mM DTT was added to *Cr*aCRY samples and they were illuminated for 15 min at 450 nm wavelength and repeatedly illuminated for 5 s at 450 nm wavelength followed by 20 s without illumination prior HDX. The dark state of *Cr*aCRY was facilitated by covering all light-transmitting vessels with aluminum foil. HDX-MS was essentially carried out as described previously^32–34^ aided by a robotic two-arm autosampler (LEAP Technologies). 7.5 µl (60 µM) CRY were diluted with 67.5 µl of D_2_O-containing buffer (10 mM Tris, 100 mM sodium chloride, pD 7.8) and incubated for 10, 95, 1000 or 10000 s at 25 °C. H/D exchange was stopped by mixing 55 µl of the reaction with an equal volume of quench buffer (400 mM KH_2_PO_4_/H_3_PO_4_, 2 M guanidine-HCl, pH 2.2) kept at 1 °C and immediately injected into an ACQUITY UPLC M-class system with HDX technology (*Waters*)^33^. *Cr*aCRY was digested with immobilized pepsin at 12 °C in water +0.1% (v/v) formic acid at a flow rate of 100 µl/min and the resulting peptides trapped on a C18 column at 0.5 °C. After 3 minutes, the C18 trap column was placed in line with an ACQUITY UPLC BEH C18 1.7 µm 1.0 × 100 mm column (Waters) and the peptides separated at 0.5 °C with a gradient of water +0.1% (v/v) formic acid (eluent A) and acetonitrile +0.1% (v/v) formic acid (eluent B) at 30 µL/min flow rate as follows: 0–7 min/95–65% A, 7–8 min/65–15% A, 8–10 min/15% A, 10–11 min/5% A, 11–16 min/95% A. Mass spectra were recorded on a G2-Si HDMS mass spectrometer (Waters) in High Definition MS (HDMS) positive ion mode. [Glu1]-fibrinopeptide B (Waters) was used for lock-mass correction. Non-deuterated samples of *Cr*aCRY were prepared similarly employing non-deuterated buffer (10 mM Tris, 100 mM sodium chloride, pH 7.8). Here, mass spectra were acquired in Enhanced High Definition MS (HDMS^E^) positive ion mode^32,34^ Between samples, the immobilized pepsin was washed three times with 80 μl of 4% (v/v) acetonitrile and 0.5 M guanidine hydrochloride. All measurements were performed in triplicates. Peptide identification and assignment of deuterium incorporation was done using the PLGS and DynamX 3.0 software (*Waters*), respectively, as described elsewhere^35,36^. Statistically significant changes in deuterium uptake were certified by using a two-sided t-test with a 98% confidence interval (Supplementary Tables S3–S4). The resulting differences in deuterium uptake were mapped onto the *Cr*aCRY∆CTE structure (PDB: 6FN0) and figures were created using PYMOL 2.0.6 (*DeLano Scientific*).

## Supplementary information

Supplementary Figures and Tables
