# Supplementary material for: Structural changes within the bifunctional cryptochrome/photolyase CraCRY upon blue light excitation
Source: Sci Rep. 2019 Jul 9;9:9896. doi: 10.1038/s41598-019-45885-7 (PMC6616342; doi:10.1038/s41598-019-45885-7)
Supplement: Supplementary file 1 — Supplementary Figures and Tables [file 41598_2019_45885_MOESM1_ESM.docx]

**Structural changes within the bifunctional cryptochrome/photolyase *Cr*aCRY upon blue light excitation**

Sophie Franz-Badur^1^, Alexander Penner^1^, Simon Straß^1,2^, Silke von Horsten^1^, Uwe Linne^1,3^ and Lars-Oliver Essen^1,3^*

^1^ Unit for Structural Biochemistry, Department of Chemistry, Philipps University Marburg, Hans-Meerwein Straße 4, 35032 Marburg, Germany

^2^ Synovo GmbH, Paul-Ehrlich-Straße 15, 72076 Tübingen, Germany

^3^ LOEWE Center of Synthetic Microbiology, Philipps University Marburg, Hans-Meerwein Straße 4, 35032 Marburg, Germany

* Corresponding Author: Tel: +49 6421/28-22032; Fax: +49 6421/28-22012. Email: essen@chemie.uni-marburg.de

**Supplementary Figures**

**Supplementary Figure S1.** **UV/Vis absorption spectra of the samples before analytical size exclusion.**

**
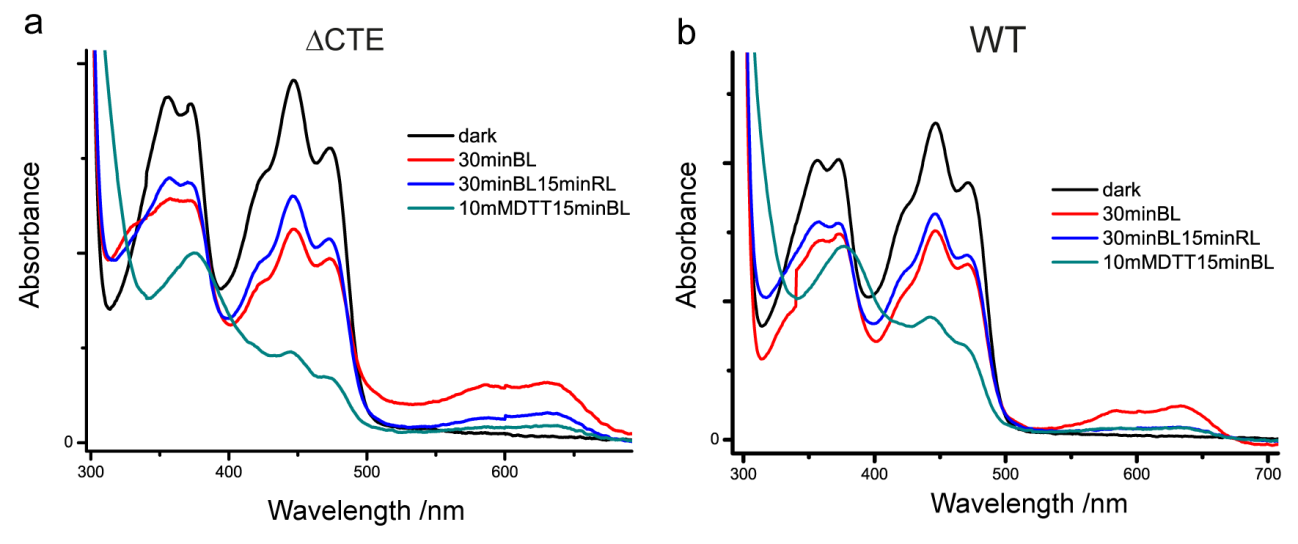
**

**Supplementary Figure S2. Illumination device for automated HDX-MS of light-sensitive proteins. a)** Schematic representation of the light chamber we designed. P1, P2 and P3 are positions of different sample holders, which can be illuminated with three different wavelengths. The 2600 mAh battery can be charged via miniUSB. **b)** Picture of the light chamber. It works completely independently and fits perfectly in the two arm robotic autosampler system (*LEAP Technologies*). **c)** Diagram of the position of the buttons to activate the different LEDs. The buttons can be switched to either the plus or minus position. Depending on the LEDs that should be activated the buttons should be in the positions as shown in the diagram. The illumination interval can be regulated over button 5. If button 5 is switched to the plus position, the LED will be on for 5 s and off for 20 s. In the minus position the interval will be 30 s of light followed by 270 s of dark.


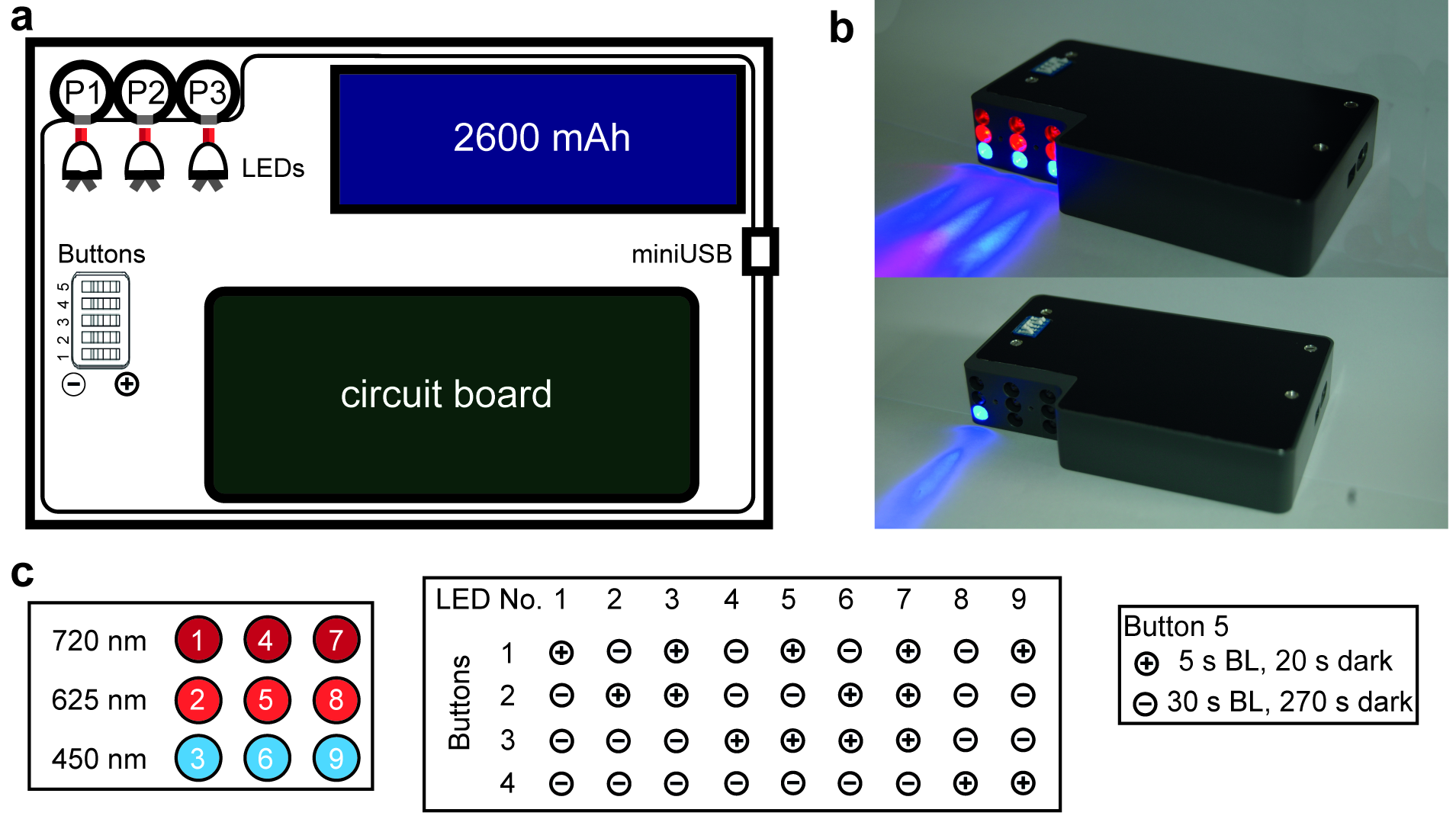


**Supplementary Figure S3. Difference map of the relative fractional uptake for the FAD_OX_ (dark) state WT minus ∆CTE.** Selected peptides were marked with either a plus (|t|>1.94) or minus (|t|<1.94).


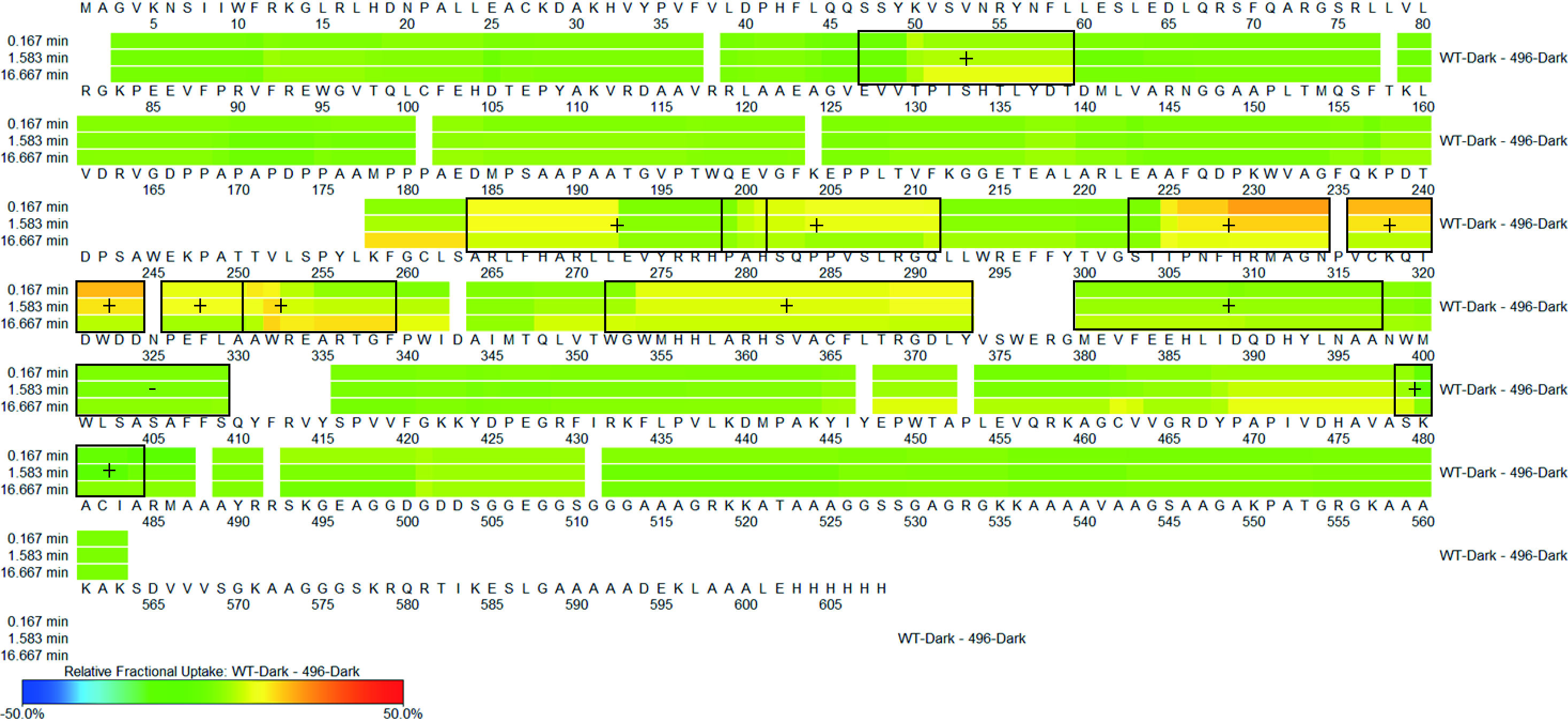


**Supplementary Figure S4. Difference map of the relative fractional uptake for ∆CTE (∆496) FADH^−^ (light) minus FAD_OX_ (dark).** Selected peptides were marked with either a plus (|t|>1.94) or minus (|t|<1.94).

**
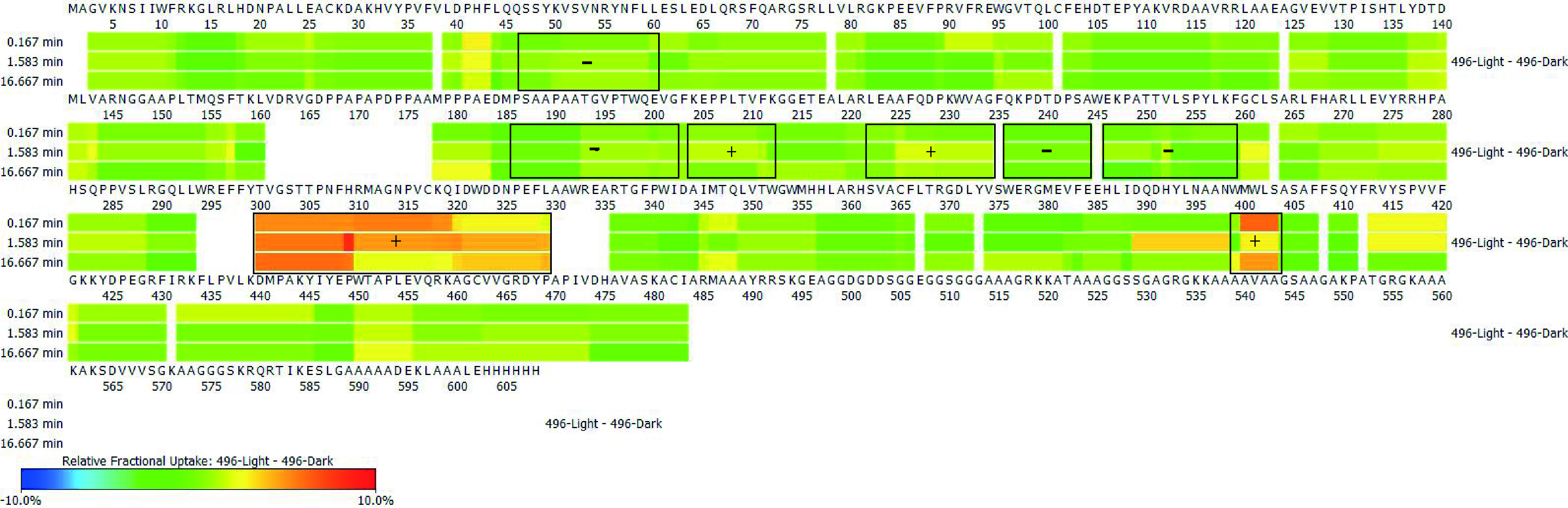
**

**Supplementary Tables**

**Supplementary Table S1.** **Analytical size exclusion chromatography (SEC) of *Cr*aCRY.** Apparent SEC masses were calculated by using the column’s calibration curve (lg MM=‑0.15642∙x_elute_+3.29765; MM in kDa). To estimate the distribution between monomeric and dimer-like species calculated molecular masses (MM) of 66.3 kDa and 57.7 kDa were used for *Cr*aCRY WT and the ∆CTE variant, respectively. The dimer-like fraction was calculated by adding up the peak integrals and calculating the percentage of the dimer-like peak of the total area.

|  | **„Dimer“**  **A_280nm_**  /mL | **Monomer**  **A_280nm_**  /mL | **“Dimer”**  **MM_app._**  /kDa | **Monomer**  **MM_app._**  /kDa | **%_Dimer_**  **A_280nm_** | **%_Dimer_**  **A_450nm_** | **%_Dimer_**  **A_370nm_** | **%^450nm/370nm^ / %^280nm^** |
| --- | --- | --- | --- | --- | --- | --- | --- | --- |
| **∆CTE** |  |  |  |  |  |  |  |  |
| dark | 8.48 | 9.63 | 94 | 62 | 4 | 0 | n.d. | 0.0 |
| 30 min BL | 8.40 | 9.53 | 96 | 64 | 23 | 29 | n.d. | 1.3 |
| BL+RL | 8.59 | 9.66 | 90 | 61 | 28 | n.d. | 28 | 1.0 |
| DTT+BL | 8.51 | 9.66 | 93 | 61 | 7 | n.d. | 0 | 0.0 |
|  |  |  |  |  |  |  |  |  |
| **WT** |  |  |  |  |  |  |  |  |
| dark | 8.05 | 8.60 | 109 | 90 | 4 | 0 | n.d. | 0.0 |
| 30 min BL | 8.10 | 8.60 | 107 | 90 | 12 | 14 | n.d. | 1.2 |
| BL+RL | 8.08 | 8.64 | 108 | 88 | 18 | n.d. | 21 | 1.2 |
| DTT+BL | 8.10 | 8.70 | 107 | 87 | 4 | n.d. | 0 | 0.0 |

**Supplementary Table S2. Selected HDX peptides of *Cr*aCRY with raw values and standard deviations (SD).** Mean relative uptake for WT and ∆CTE for an incubation time of 10 s as well as the percentage uptake.

| **Start** | **End** | **MaxD** | **Protein** | **State** | **Uptake /Da** | **Uptake SD /Da** | **Uptake /%** |
| --- | --- | --- | --- | --- | --- | --- | --- |
| 47 | 59 | 12 | ∆CTE | FAD_OX_ | 3.6 | 0.1 | 0.83 |
| 47 | 59 | 12 | ∆CTE | FADH^−^ | 3.6 | 0.0 | 0.15 |
| 47 | 59 | 12 | WT | FAD_OX_ | 4.5 | 0.1 | 2.37 |
| 47 | 59 | 12 | WT | FADH^−^ | 4.4 | 0.1 | 1.82 |
| 184 | 202 | 15 | ∆CTE | FAD_OX_ | 5.3 | 0.1 | 0.48 |
| 184 | 202 | 15 | ∆CTE | FADH^−^ | 5.3 | 0.0 | 0.63 |
| 184 | 202 | 15 | WT | FAD_OX_ | 5.7 | 0.0 | 2.52 |
| 184 | 202 | 15 | WT | FADH^−^ | 5.7 | 0.0 | 2.43 |
| 199 | 211 | 10 | ∆CTE | FAD_OX_ | 3.0 | 0.0 | 0.82 |
| 199 | 211 | 10 | ∆CTE | FADH^−^ | 3.0 | 0.0 | 1.93 |
| 199 | 211 | 10 | WT | FAD_OX_ | 3.7 | 0.0 | 3.65 |
| 199 | 211 | 10 | WT | FADH^−^ | 3.6 | 0.0 | 4.85 |
| 223 | 234 | 10 | ∆CTE | FAD_OX_ | 1.6 | 0.0 | 0.38 |
| 223 | 234 | 10 | ∆CTE | FADH^−^ | 1.7 | 0.1 | 0.30 |
| 223 | 234 | 10 | WT | FAD_OX_ | 3.1 | 0.0 | 2.45 |
| 223 | 234 | 10 | WT | FADH^−^ | 3.0 | 0.1 | 1.96 |
| 235 | 244 | 7 | ∆CTE | FAD_OX_ | 0.7 | 0.1 | 0.84 |
| 235 | 244 | 7 | ∆CTE | FADH^−^ | 0.6 | 0.0 | 2.69 |
| 235 | 244 | 7 | WT | FAD_OX_ | 2.1 | 0.1 | 3.04 |
| 235 | 244 | 7 | WT | FADH^−^ | 2.1 | 0.0 | 8.94 |
| 245 | 250 | 4 | ∆CTE | FAD_OX_ | 1.1 | 0.0 | 1.13 |
| 245 | 250 | 4 | ∆CTE | FADH^−^ | 1.1 | 0.0 | 2.51 |
| 245 | 250 | 4 | WT | FAD_OX_ | 1.5 | 0.0 | 2.49 |
| 245 | 250 | 4 | WT | FADH^−^ | 1.6 | 0.0 | 8.39 |
| 245 | 259 | 12 | ∆CTE | FAD_OX_ | 1.5 | 0.1 | 0.41 |
| 245 | 259 | 12 | ∆CTE | FADH^−^ | 1.5 | 0.0 | 1.56 |
| 245 | 259 | 12 | WT | FAD_OX_ | 3.1 | 0.0 | 2.66 |
| 245 | 259 | 12 | WT | FADH^−^ | 3.1 | 0.0 | 3.99 |
| 272 | 293 | 18 | ∆CTE | FAD_OX_ | 2.1 | 0.0 | 0.64 |
| 272 | 293 | 18 | ∆CTE | FADH^−^ | 2.2 | 0.0 | 1.84 |
| 272 | 293 | 18 | WT | FAD_OX_ | 4.8 | 0.1 | 2.34 |
| 272 | 293 | 18 | WT | FADH^−^ | 4.8 | 0.1 | 5.75 |
| 299 | 317 | 16 | ∆CTE | FAD_OX_ | 2.1 | 0.0 | 1.25 |
| 299 | 317 | 16 | ∆CTE | FADH^−^ | 2.9 | 0.0 | 0.86 |
| 299 | 317 | 16 | WT | FAD_OX_ | 2.2 | 0.0 | 1.86 |
| 299 | 317 | 16 | WT | FADH^−^ | 2.7 | 0.1 | 3.00 |
| 318 | 328 | 9 | ∆CTE | FAD_OX_ | 1.8 | 0.1 | 0.56 |
| 318 | 328 | 9 | ∆CTE | FADH^−^ | 2.1 | 0.1 | 0.54 |
| 318 | 328 | 9 | WT | FAD_OX_ | 1.9 | 0.0 | 2.44 |
| 318 | 328 | 9 | WT | FADH^−^ | 2.0 | 0.0 | 2.23 |
| 399 | 404 | 5 | ∆CTE | FAD_OX_ | 0.6 | 0.0 | 0.71 |
| 399 | 404 | 5 | ∆CTE | FADH^−^ | 0.9 | 0.0 | 3.82 |
| 399 | 404 | 5 | WT | FAD_OX_ | 0.4 | 0.0 | 3.94 |
| 399 | 404 | 5 | WT | FADH^−^ | 0.5 | 0.0 | 9.73 |

**Supplementary Table S3. Fractional uptakes for the FAD_OX_ and FADH^−^ states of WT vs. ∆CTE.** Statistically significant changes in deuterium uptake from selected peptides between ∆CTE and WT were certified by using a two-sided t-test with a 98 % (|t|>1.94).

| Start | End | Protein | State | s^2^ | s | t | Significant? |
| --- | --- | --- | --- | --- | --- | --- | --- |
| 47 | 59 | ∆CTE | FAD_OX_ | 7.29∙10^-3^ | 8.54∙10^-2^ | -11.9 | + |
| 47 | 59 | WT | FAD_OX_ |  |  |  |  |
| 47 | 59 | ∆CTE | FADH^−^ | 2.56∙10^-3^ | 5.06∙10^-2^ | -19.4 | + |
| 47 | 59 | WT | FADH^−^ |  |  |  |  |
| 184 | 202 | ∆CTE | FAD_OX_ | 1.73∙10^-3^ | 4.16∙10^-2^ | -13.8 | + |
| 184 | 202 | WT | FAD_OX_ |  |  |  |  |
| 184 | 202 | ∆CTE | FADH^−^ | 1.12∙10^-3^ | 3.35∙10^-2^ | -14.6 | + |
| 184 | 202 | WT | FADH^−^ |  |  |  |  |
| 199 | 211 | ∆CTE | FAD_OX_ | 1.09∙10^-4^ | 1.04∙10^-2^ | -79.9 | + |
| 199 | 211 | WT | FAD_OX_ |  |  |  |  |
| 199 | 211 | ∆CTE | FADH^−^ | 8.94∙10^-4^ | 2.99∙10^-2^ | -25.8 | + |
| 199 | 211 | WT | FADH^−^ |  |  |  |  |
| 223 | 234 | ∆CTE | FAD_OX_ | 5.51∙10^-4^ | 2.35∙10^-2^ | -76.2 | + |
| 223 | 234 | WT | FAD_OX_ |  |  |  |  |
| 223 | 234 | ∆CTE | FADH^−^ | 2.95∙10^-3^ | 5.43∙10^-2^ | -30.4 | + |
| 223 | 234 | WT | FADH^−^ |  |  |  |  |
| 235 | 244 | ∆CTE | FAD_OX_ | 3.28∙10^-3^ | 5.73∙10^-2^ | -30.0 | + |
| 235 | 244 | WT | FAD_OX_ |  |  |  |  |
| 235 | 244 | ∆CTE | FADH^−^ | 9.15∙10^-4^ | 3.02∙10^-2^ | -60.6 | + |
| 235 | 244 | WT | FADH^−^ |  |  |  |  |
| 245 | 250 | ∆CTE | FAD_OX_ | 5.99∙10^-4^ | 2.45∙10^-2^ | -21.0 | + |
| 245 | 250 | WT | FAD_OX_ |  |  |  |  |
| 245 | 250 | ∆CTE | FADH^−^ | 2.42∙10^-4^ | 1.56∙10^-2^ | -35.4 | + |
| 245 | 250 | WT | FADH^−^ |  |  |  |  |
| 245 | 259 | ∆CTE | FAD_OX_ | 3.80∙10^-3^ | 6.16∙10^-2^ | -33.0 | + |
| 245 | 259 | WT | FAD_OX_ |  |  |  |  |
| 245 | 259 | ∆CTE | FADH^−^ | 1.36∙10^-3^ | 3.68∙10^-2^ | -53.2 | + |
| 245 | 259 | WT | FADH^−^ |  |  |  |  |
| 272 | 293 | ∆CTE | FAD_OX_ | 2.71∙10^-3^ | 5.21∙10^-2^ | -63.5 | + |
| 272 | 293 | WT | FAD_OX_ |  |  |  |  |
| 272 | 293 | ∆CTE | FADH^−^ | 2.01∙10^-3^ | 4.48∙10^-2^ | -70.5 | + |
| 272 | 293 | WT | FADH^−^ |  |  |  |  |
| 299 | 317 | ∆CTE | FAD_OX_ | 6.89∙10^-4^ | 2.62∙10^-2^ | -7.47 | + |
| 299 | 317 | WT | FAD_OX_ |  |  |  |  |
| 299 | 317 | ∆CTE | FADH^−^ | 2.56∙10^-3^ | 5.05∙10^-2^ | 5.82 | + |
| 299 | 317 | WT | FADH^−^ |  |  |  |  |
| 318 | 328 | ∆CTE | FAD_OX_ | 3.14∙10^-3^ | 5.60∙10^-2^ | -1.75 | - |
| 318 | 328 | WT | FAD_OX_ |  |  |  |  |
| 318 | 328 | ∆CTE | FADH^−^ | 2.21∙10^-3^ | 4.70∙10^-2^ | 1.56 | - |
| 318 | 328 | WT | FADH^−^ |  |  |  |  |
| 399 | 404 | ∆CTE | FAD_OX_ | 5.06∙10^-4^ | 2.25∙10^-2^ | 11.8 | + |
| 399 | 404 | WT | FAD_OX_ |  |  |  |  |
| 399 | 404 | ∆CTE | FADH^−^ | 7.10∙10^-4^ | 2.66∙10^-2^ | 18.0 | + |
| 399 | 404 | WT | FADH^−^ |  |  |  |  |

**Supplementary Table S4. Fractional uptakes for WT and ∆CTE in the FAD_OX_ state vs. the FADH^−^ state.** Statistically significant changes in deuterium uptake from selected peptides between FAD_OX_ and FADH^−^ were certified by using a two-sided t-test with a 98 % (|t|>1.94).

| Start | End | Protein | State | s^2^ | s | t | Significant? |
| --- | --- | --- | --- | --- | --- | --- | --- |
| 47 | 59 | ∆CTE | FAD_OX_ | 3.08∙10^-3^ | 5.55∙10^-2^ | 0.88 | - |
| 47 | 59 | ∆CTE | FADH^−^ |  |  |  |  |
| 47 | 59 | WT | FAD_OX_ | 6.78∙10^-3^ | 8.23∙10^-2^ | 1.04 | - |
| 47 | 59 | WT | FADH^−^ |  |  |  |  |
| 184 | 202 | ∆CTE | FAD_OX_ | 1.50∙10^-3^ | 3.87∙10^-2^ | -1.27 | - |
| 184 | 202 | ∆CTE | FADH^−^ |  |  |  |  |
| 184 | 202 | WT | FAD_OX_ | 1.36∙10^-3^ | 3.68∙10^-2^ | 0.10 | - |
| 184 | 202 | WT | FADH^−^ |  |  |  |  |
| 199 | 211 | ∆CTE | FAD_OX_ | 6.97∙10^-5^ | 8.35∙10^-3^ | 4.40 | + |
| 199 | 211 | ∆CTE | FADH^−^ |  |  |  |  |
| 199 | 211 | WT | FAD_OX_ | 9.33∙10^-4^ | 3.05∙10^-2^ | 3.21 | + |
| 199 | 211 | WT | FADH^−^ |  |  |  |  |
| 223 | 234 | ∆CTE | FAD_OX_ | 1.69∙10^-3^ | 4.11∙10^-2^ | -2.38 | + |
| 223 | 234 | ∆CTE | FADH^−^ |  |  |  |  |
| 223 | 234 | WT | FAD_OX_ | 1.81∙10^-3^ | 4.25∙10^-2^ | 0.86 | - |
| 223 | 234 | WT | FADH^−^ |  |  |  |  |
| 235 | 244 | ∆CTE | FAD_OX_ | 1.86∙10^-3^ | 4.31∙10^-2^ | 1.51 | - |
| 235 | 244 | ∆CTE | FADH^−^ |  |  |  |  |
| 235 | 244 | WT | FAD_OX_ | 2.34∙10^-3^ | 4.84∙10^-2^ | -1.01 | - |
| 235 | 244 | WT | FADH^−^ |  |  |  |  |
| 245 | 250 | ∆CTE | FAD_OX_ | 4.06∙10^-4^ | 2.02∙10^-2^ | 0.61 | - |
| 245 | 250 | ∆CTE | FADH^−^ |  |  |  |  |
| 245 | 250 | WT | FAD_OX_ | 4.35∙10^-4^ | 2.09∙10^-2^ | -1.17 | - |
| 245 | 250 | WT | FADH^−^ |  |  |  |  |
| 245 | 259 | ∆CTE | FAD_OX_ | 4.16∙10^-3^ | 6.45∙10^-2^ | -0.76 | - |
| 245 | 259 | ∆CTE | FADH^−^ |  |  |  |  |
| 245 | 259 | WT | FAD_OX_ | 9.87∙10^-4^ | 3.14∙10^-2^ | 0.78 | - |
| 245 | 259 | WT | FADH^−^ |  |  |  |  |
| 272 | 293 | ∆CTE | FAD_OX_ | 1.01∙10^-3^ | 3.17∙10^-2^ | -2.70 | + |
| 272 | 293 | ∆CTE | FADH^−^ |  |  |  |  |
| 272 | 293 | WT | FAD_OX_ | 3.72∙10^-3^ | 6.10∙10^-2^ | 1.00 | - |
| 272 | 293 | WT | FADH^−^ |  |  |  |  |
| 299 | 317 | ∆CTE | FAD_OX_ | 1.12∙10^-3^ | 3.35∙10^-2^ | -29.6 | + |
| 299 | 317 | ∆CTE | FADH^−^ |  |  |  |  |
| 299 | 317 | WT | FAD_OX_ | 2.12∙10^-3^ | 4.61∙10^-2^ | -10.9 | + |
| 299 | 317 | WT | FADH^−^ |  |  |  |  |
| 318 | 328 | ∆CTE | FAD_OX_ | 3.70∙10^-3^ | 6.08∙10^-2^ | -5.44 | + |
| 318 | 328 | ∆CTE | FADH^−^ |  |  |  |  |
| 318 | 328 | WT | FAD_OX_ | 1.65∙10^-3^ | 4.07∙10^-2^ | -3.92 | + |
| 318 | 328 | WT | FADH^−^ |  |  |  |  |
| 399 | 404 | ∆CTE | FAD_OX_ | 1.04∙10^-3^ | 3.22∙10^-2^ | -11.9 | + |
| 399 | 404 | ∆CTE | FADH^−^ |  |  |  |  |
| 399 | 404 | WT | FAD_OX_ | 1.77∙10^-4^ | 1.33∙10^-2^ | -12.7 | + |
| 399 | 404 | WT | FADH^−^ |  |  |  |  |
